# Data-driven analysis of climate impact on tomato and apple prices using machine learning

**DOI:** 10.1016/j.heliyon.2024.e41478

**Published:** 2024-12-25

**Authors:** Sunghyun Yoon, Tae-Hwa Kim, Dong Sub Kim

**Affiliations:** a Department of Artificial Intelligence, Kongju National University, Cheonan, 31080, Republic of Korea; b Department of Community Development, Kongju National University, Yesan, 32439, Republic of Korea; c Department of Horticulture, Kongju National University, Yesan, 32439, Republic of Korea

**Keywords:** Tomato and apple prices, Climate change, Cloud amount, LSTM, Time delay

## Abstract

Machine learning has been used in various areas, but there are few studies on price prediction for agricultural products. Here, a machine learning technique for the price prediction of tomato and apple fruits was attempted based on environment and price data for 12 years. The goal of this study is to discover 1) how much can we accurately predict the product prices with the environmental factors and 2) how much each environmental factor affects to the product prices. This study assumes that the environmental factors directly affect crop growth and thus indirectly determine fruit production and accompanying price. In addition, it is assumed that there are two kinds of time lags, between the change in the environmental factors and their effects on the crop growth, and between the change in the crop growth and its effect on the price. In the process, machine learning techniques were used instead of econometric models commonly used in agricultural economics. The relationship between the environmental factors and fruit price with varying time lags in data-driven manner using long short-term memory (LSTM) was modeled in this study. The study empirically revealed that there are suitable time lags between the environmental factors and fruit price in the price prediction, and taking these time lags into the prediction improves the accuracy. Moreover, the importance of each of the environmental factors on the price using shapely additive explanations (SHAP) was demonstrated though this study, which assists the decision-making process in agriculture against the climate change.

## 1 Introduction

The price of agricultural products depends on a lot of factors that affect the entire process from the production to the consumption, such as cultivation environments, crop yield, consumer preferences, and changes in macroeconomics like inflation, international trade. Understanding the factors affecting agricultural product prices is crucial for promoting economic stability at the national level, ensuring food security, and enhancing market efficiency. Therefore, many studies have been performed to investigate the impact of the various factors on the agricultural product price [[1], [2], [3], [4]].

Agricultural product prices generally exhibit competitive, seasonal, and volatile characteristics. These features are largely influenced by the impact of climate and pests during the production process, as well as the perishable nature of agricultural products, which incurs significant costs in logistics such as transportation, storage, and packaging. Additionally, the inelastic demand for prices further contributes to the volatility of agricultural product prices. The various factors that influence demand and supply in the market are constantly changing, leading to continuous fluctuations in prices. Individual economic entities are exposed to the risks and uncertainties of price since they cannot predict the direction or level of future price changes. This exposure poses challenges in economic decision-making, creating difficulties in navigating the uncertainties associated with price movements [1,5].

Recently, there has been a frequent occurrence of price issues in agricultural products in South Korea, and there is a growing number of items experiencing significant fluctuations in agricultural product shipment volumes. This increase is considered to contribute the characteristics of agricultural products, where production quantity and quality are influenced by natural conditions such as adequate sunlight, temperature, humidity, and moisture. Due to these characteristics, cases influenced by the intensifying 'abnormal climate' phenomena like droughts, floods, frost, and heatwaves have become more prevalent. Due to unpredictable environmental conditions, there is an increasing occurrence of crops nearing harvest being damaged, causing disruptions in shipments and influencing agricultural product prices [6].

Climate change has become a threatening factor to secure stable food supplies, a fundamental condition for human survival, and is significantly impacting agriculture. Recently, in Korea, climate change has led to shifts in cultivation areas, with primary production zones moving northward, causing changes in arable land [7]. This shift is posing challenges to maintaining both the quantity and quality of agricultural product yields. Additionally, the emergence of new pests and diseases in the altered climate is making agricultural production more challenging [[8], [9], [10]]. As the cultivation environment is changing dramatically due to climate change, various models and techniques are being introduced to predict prices using air temperature, relative humidity, etc. as the factors.

[11] demonstrated the price results for the coarse grains, oil seeds, rice, sugar, and wheat according to climate change scenarios. A few models for the sugar displayed price decline in the selected scenarios but price raising was the trend. High air temperature reduced yield of various vegetables, and this may temporarily raise the prices and become a major factor that enhances the price fluctuations [12]. On the other hand, if the period until the environmental factors affects the price is informed, predicting the price may become more accurate. But the study requires a lot of data during long period.

Analyzing the impact of environmental factors on agricultural product prices is a crucial task in understanding and stabilizing agricultural production and supply. Through this analysis, it is possible to minimize uncertainty in the agricultural product market, stabilize production. This, in turn, aids in designing policies that address the challenges posed by climate change in agriculture. Over the years, various studies have been conducted using econometric models to analyze the impact of environmental factors on agricultural product prices. Most models in the field of deep learning have sufficiently high complexity for modeling complex patterns inherent in data [13]. Thus, if the underlying relationships between input-output variables have actually quite complex, it is difficult to model those complex patterns. Whereas, deep learning techniques could provide outstanding performances compared to traditional techniques in various domains, if the sufficient amount of data are provided and the methodologies used for modeling have the appropriate inductive biases. Moreover, sufficiently high-dimensional input data containing various information are given, deep learning techniques could provide opportunities to discover useful insights inherent in data but not discovered yet [14]. Among various neural network-based models, long short-term memory (LSTM) was adopted, which is a popular method to capture the time dependency in sequential data. This technology is used in agriculture in a variety of ways such as estimation of evapotranspiration and soil water content [15], estimation of the number of fruits and the size of the fruits [16], and soil temperature prediction [17]. However, to date, there have been only a few studies that have utilized artificial intelligence to analyze the impact of various environmental factors on agricultural product prices (Table 1). Recently, the public data portal service was launched, making it possible to use big data about the information and conduct precision investigations. Here, for automatically and systemically analyzing big data, data-driven modeling techniques are recommended. Strategies using artificial intelligence are the most attractive at the moment [18,19].

**Table 1:** Previous studies on environmental factors, price, and machine learning for tomatoes and apples.

Crop	Environmental factor	Price	Machine learning	Reference
Tomato	O	O	X	[20]
	O	X	O	[21]
	X	O	O	[22,23]
	X	X	O	[24]
Apple	O	O	X	[25]
	O	X	O	[26]
	X	O	O	[27]
	X	X	O	[28,29]

Because many fruits are harvested continuously after being cultivated for a long period, environment conditions during the cultivation inevitably affect price directly and indirectly. For example, higher air temperature due to the climate change may exhibit severe yield production [30]. In particular, the heat stress during reproductive stages resulted in considerable loss of yield because floret fertility [31,32] and the duration of seed filling [33] is reduced by the heat stress. This reduction in fruit production may affect prices based on the laws of supply and demand. In South Korea, tomato cultivation is mainly done in greenhouses while apple cultivation is mostly done in open field (KOSIS). Thus, the two crops are expected to have differences in growth and production in response to climate change. Moreover, because tomato and apple fruits are the most globally cultivated fruits in the world [34], therefore, the study on the two fruits should take precedence.

Previous studies have focused on improving the accuracy of agricultural product price prediction according to environmental factors [4,35]. However, environmental factors certainly affect the price of agricultural products, but since there is a time lag between the environment conditions and fruit production, there is a high probability that the impact of environmental factors on the price may be delayed during a certain period. Taking in to account the duration of environmental factors in understanding the impact on agricultural product prices is important as it can be used fundamental information for formulating climate change-responsive agricultural production strategies and designing relevant polices.

Climate change makes it more difficult to predict prices of agricultural products. This phenomenon means that farmers may not be guaranteed profits and consumers may purchase expensive agricultural products. The purpose of this study is to investigate how long the time lag between the change in environmental factors and its effects on the prices of tomato and apple, and to evaluate the possibility of the price prediction based on the environment data using machine learning. This contributes to ensuring a stable supply of agricultural products and providing price stability for consumers. It allows for exploring ways in which the agricultural sector can develop sustainably. Above all, through such analysis, design agricultural policies to respond to climate change can be designed. The structure of this study consists of introduction, methodology (environmental and price data collection and statistical analysis for time delay model architecture), results (performance for price prediction and explanatory power of price), discussion, and conclusion.

## 2 Methodology

### 2.1 Data collection

The environmental data, exchange volume data, and wholesale price data used in this study are downloaded from KREI OASIS (https://oasis.krei.re.kr). The attributes and statistics from the data is provided in Table 2, Table 3, respectively. The environmental and exchange volume data correspond to independent variables, and the price data corresponds to the dependent variable.

**Table 2:** List of attributes of the environmental, exchange volume, and wholesale price data.

	Name	Description	Unit
Environmental factor (Explanatory variable)	Temp_mean	Daily mean temperature	^o^C
	Temp_max	Daily maximum temperature	^o^C
	Temp_min	Daily minimum temperature	^o^C
	Temp_last_year	Mean temperature at the corresponding day last year.	^o^C
	Temp_avg_tear	Mean temperature over the last 30 years	^o^C
	Prec_mean	Daily mean precipitation	mm
	Prec_last_year	Mean precipitation at the corresponding day last year.	mm
	Prec_avg_year	Mean precipitation over the last 30 years	mm
	Sunsh_mean	Daily mean sunshine hours	hr
	Sunsh_last_year	Mean sunshine hours at the corresponding day last year	hr
	Sunsh_avg_year	Mean sunshine hours over the last 30 years	hr
	Solar_mean	Daily mean quantity of solar radiation	MJ m^−2^
	Solar_last_year	Mean quantity of solar radiation at the corresponding day last year	MJ m^−2^
	Solar_avg_year	Mean quantity of solar radiation over the last 30 years	MJ m^−2^
	Humid_mean	Daily mean humidity	%
	Humid_last_year	Mean humidity at the corresponding day last year	%
	Humid_avg_year	Mean humidity at the corresponding day last year	%
	Cloud_mean	Daily mean cloud amount	1/10
	Cloud_last_year	Mean amount of cloud at the corresponding day last year	1/10
	Cloud_avg_year	Mean amount of cloud over the last 30 yeras	1/10
	Snow_mean	Daily mean amount of snowfall	cm
	Snow_last_year	Mean amount of snowfall at the corresponding day last year	cm
	Snow_avg_year	Mean amount of snowfall over the last 30 years	cm
	Wind_mean	Daily mean wind speed	m s^−1^
	Wind_last_year	Mean wind speed at the corresponding day last year	m s^−1^
	Wind_avg_year	Mean wind speed over the last 30 years	m s^−1^
Exchange volume (Optional explanatory variable)	Volume	Daily total exchange volume	EA
Fruit price (Response variable)	Price	Daily mean fruit wholesale price	₩

**Table 3:** Descriptive statistics of data used in this study.

	Air temperature	Precipitation	Sunshine	Solar radiation
Average	13.03138566	3.4118379	6.2674718	4.87283391
Maximum	29.88823529	104.9029851	12.8	11.16462687
Minimum	−11.50447761	0	0.0014493	0.30161764
Coefficient of variation	0.72797923	2.5258822	0.5257517	0.46637159

	Relative humidity	Cloud amount	Snow amount	Wind velocity

Average	68.51592105	3.17212397	0.031288001	8.11575883
Maximum	95.58507463	9.81641791	2.054411765	23.16176471
Minimum	30.60144928	0.00588235	0	4.07205882
Coefficient of variation	0.18473391	0.74328512	4.325981698	0.27544545

	Tomato price	Tomato production	Apple price	Apple production

Average	2726.076175	96.88707743	3303.80795	47.50520034
Maximum	14717	325	10966	512.8
Minimum	464	3.2	680	0
Coefficient of variation	0.480330073	0.451737501	0.416614001	1.063736595

The environmental data were 24-dimensional time-series data, consisting of the factor related to air temperature, precipitation, sunlight amount, solar radiation, relative humidity, cloud amount, snow amount, and instantaneous wind speed. All data except the air temperature were collected as average, previous year, and average year data. For the air temperatures, maximum and minimum values were additionally contained. For each day and dimension, there are several values for different regions. The average value across the regions was used. The exchange volume data is optional. To compare the effect of the volume on the price prediction, two kinds of models were built: one does not utilize the volume, the other takes the volume as input. Note that the volume data have several missing values, which means there were no trades on those days. These values were filled to zero.

For the price data, the daily average prices of two fruits were used: tomatoes and apples. There are three kinds of prices depending on the unit packs: 5, 10, and 15 kg. The average price for each day across the unit packs was used simply. There were two kinds of models according to the type of price: nominal and real-term prices. The nominal price corresponds to the price not calibrated, directly accessed from the database. The real-term price means the price compensated for the variations caused by the rate of inflation, using the producer price index (PPI), available on KOSIS (https://kosis.kr). Among several varieties, 'General' tomato data was selected and used due to no cultivar information and ‘Fuji’ apple data (*Malus domestica* Borkh. ‘Fuji’), a major cultivar in South Korean, was selected and used for this study.

All the data spans 12 years from December 1, 2011, to November 30, 2023. The models were trained spanning 9 years from December 1, 2011 to November 30, 2020. They were then evaluated spanning 3 years from December 1, 2020, to November 30, 2023. (December 2011 to November 2023). All sequences were normalized to have the zero mean and unit variance.

### 2.2 Environmental data

**Fig. 1:**
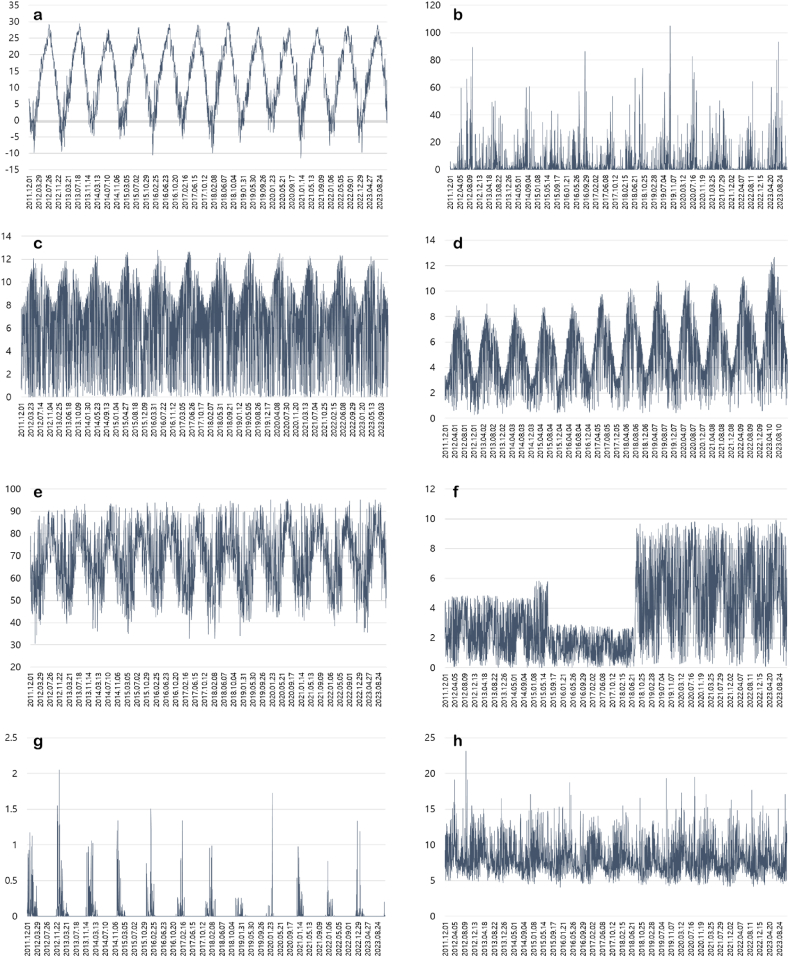
The environmental data. a: air temperature; b: precipitation; c: sunshine; d: solar radiation; e: relative humidity; f: cloud amount; g: snow amount; h: wind velocity.

**Fig. 2:**
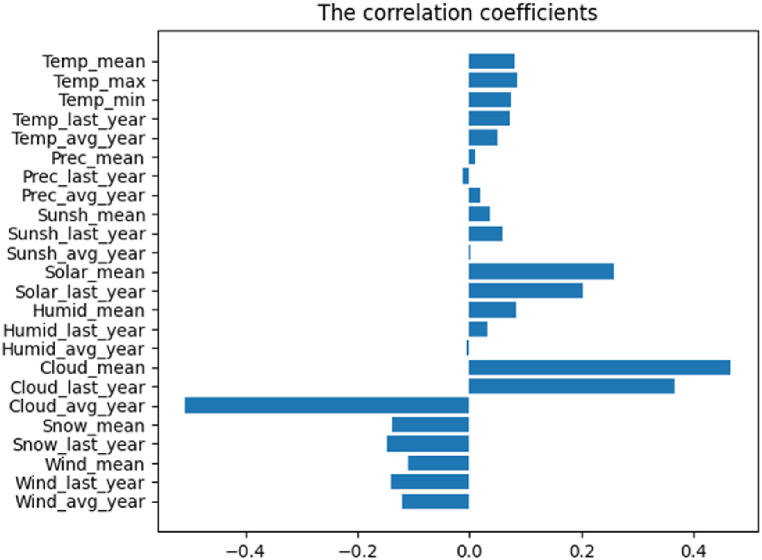
The correlation coefficients each of environmental factors to the time progress.

Generally, in South Korea, the air temperature, precipitation, sunlight amount, solar radiation, relative humidity, and cloud amount were higher in summer seasons and the snow amount and instantaneous wind speed were higher in winter seasons (Fig. 1a: air temperature; 1b: precipitation; 1c: sunshine; 1d: solar radiation; 1e: relative humidity; 1f: cloud amount; 1g: snow amount; 1h: wind velocity). Fig. 2 shows the correlation coefficients of each of environmental factors to the time progress. The time progress values to a sequence whose elements start from one and increase by one per day were set arbitrarily, i.e. [1,…,D], where D is the number of days spanning 12 years. In terms of mean value, what is noticeable is that the solar radiation and cloud amount continue to significantly increase. The cloud amount approximately doubled in 2018. The temperature and humidity have slightly increased. On the contrary, the snow amount and the maximum wind speed are gradually decreasing. The changes in precipitation and sunshine seem to be negligible.

### 2.3 Price data

**Fig. 3:**
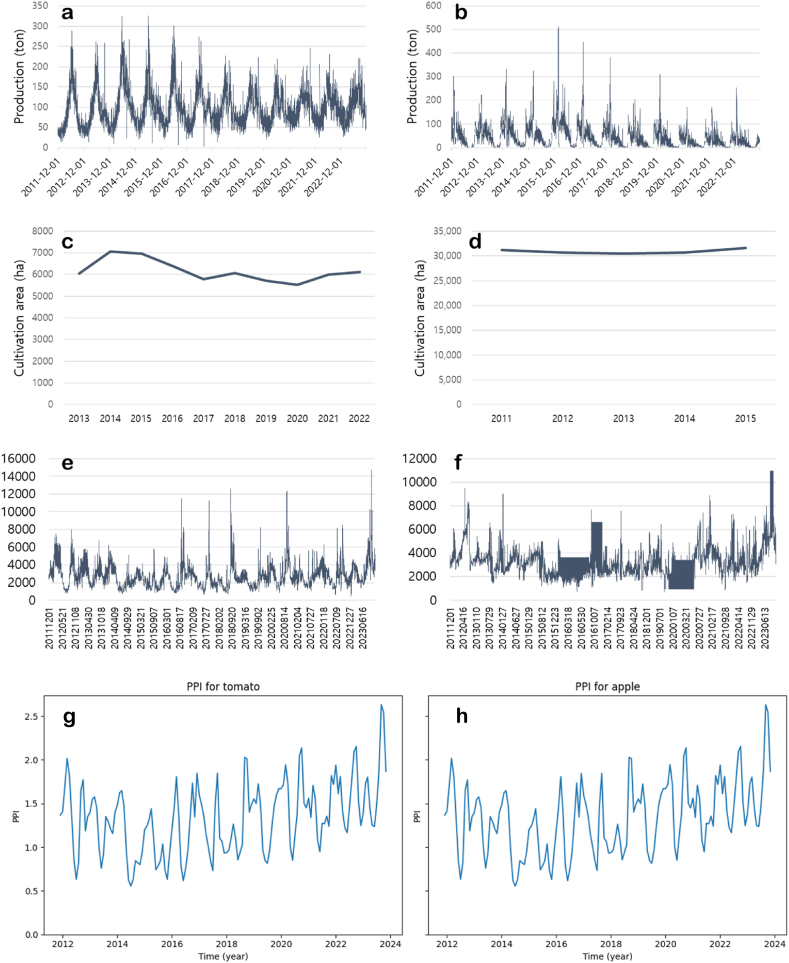
The productions (a), cultivation areas (c), nominal prices (e), and producer price indices (PPIs) (g) of tomatoes and productions (b), cultivation areas (d), nominal prices (f), and producer price indices (PPIs) (h)of apples.

Fig. 3 shows the production volume (Fig. 3a and b), cultivation area (Fig. 3c and d), nominal prices (Fig. 3e and f), and producer price indices (PPIs) (Fig. 3g and h) of tomato (Fig. 3a, 3c, 3e and 3g) and apple (Fig. 3b, 3d, 3f and 3h), respectively. The production of both tomatoes and apples were higher in summer seasons than in winter seasons. But in the case of tomatoes, the fluctuations have been narrowing, where the standard deviations of production of tomatoes and apples are 44.26 and 53.37 tons, respectively. Notice that there were 833 (16.3 %) and 1021 (20 %) missing values (i.e., no trades on those days) out of 5113 values for tomato and apple, respectively. The cultivation areas were maintained at a constant level for both tomato and apple. The nominal prices have continued to fluctuate but have been gradually rising recently. In particular, the price in 2023 was the most expensive. This result might be due to the inflations. The correlation coefficients between the nominal price and time progress were 0.048 for tomatoes and 0.064 for apples, respectively. Meanwhile, the correlation coefficients between the real-term price (i.e., compensated by the PPI and time progress were −0.336 for tomatoes and −0.236 for apples, respectively.

**Fig. 4:**
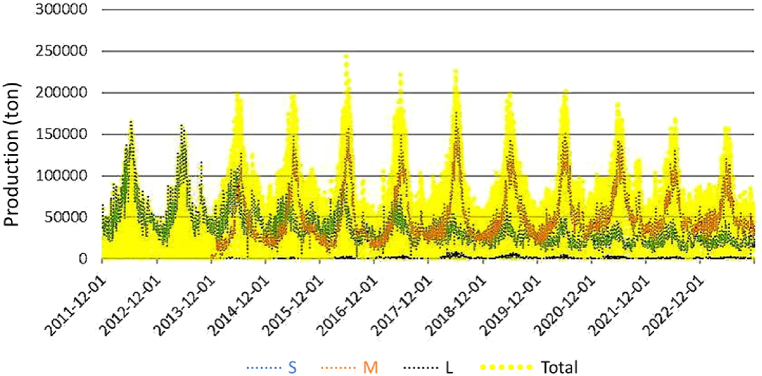
The production of various size tomatoes. S: small size; M: medium size; L: large size.

In the case of tomatoes, since the data is for all tomatoes, the production volume of other tomatoes was investigated to obtain values as close to 'General' tomato data as possible (Fig. 4). There are three types of small-sized tomatoes in Korea, and their production has been maintained at a constant level. Therefore, it is believed that the small-sized tomatoes may have an impact on the tomato prices.

### 2.4 Statistical analysis for time delay

It is reasonable to assume that there is some time gap in the optimal relationship between the weather and price patterns, because the weather on a certain day not immediately affects the price on that day. In order to preliminary confirm this assumption, the cross correlations between the sequences of weathers and volume (corresponding to the independent variables) and the sequence of price (corresponding to the dependent variable) were calculated. Let x[n] be a discrete sequence of the independent variable corresponding to a certain factor, and y[n] be that of the dependent variable. The cross correlation between x[n] and y[n] with the time delay d is calculated as in Equation (1):

(1) $$\left(x⋆y\right)\left[d\right]=\sum_{n=-\infty }^{\infty }x\left[n-d\right]y\left[n\right]=\sum_{n=-\infty }^{\infty }x\left[n\right]y\left[n+d\right]$$

where the undefined values (e.g., those with negative indices n) are considered as zero. The lager absolute value of the correlation, the more degree those sequences, x[n] and y[n], are related to each other. The cross correlation using *statsmodels* was implemented [36].

**Fig. 5:**
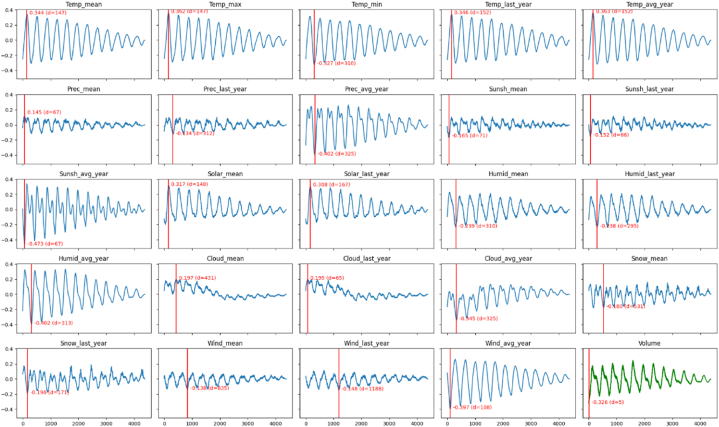
The cross correlations between the sequences of 24 environmental conditions and 1 vol value and the sequence of wholesale prices of tomato. The x-axis corresponds to the time delay d. The red vertical line represents the delay with the highest absolute correlation coefficient.

**Fig. 6:**
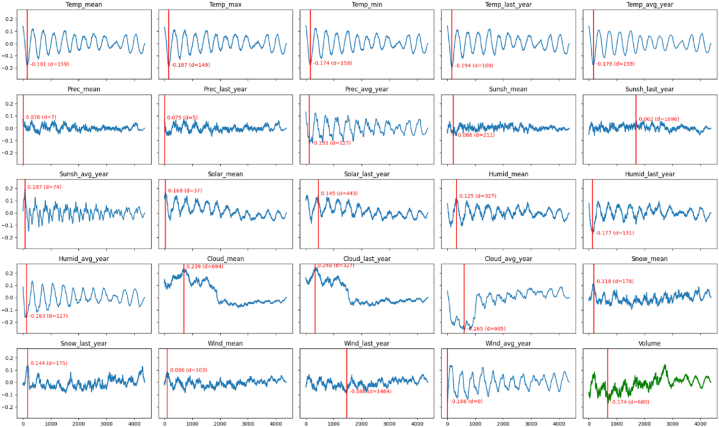
The cross correlations between the sequences of 24 environmental conditions and 1 vol value and the sequence of wholesale prices of apple. The x-axis corresponds to the time delay d. The red vertical line represents the delay with the highest absolute correlation coefficient.

Fig. 5, Fig. 6 show the cross correlations for tomato and apple, respectively. The red vertical line and accompanying annotation indicate the optimal position of d and corresponding correlation value, respectively. Except *Wind_avg* (i.e., the average of the maximum instantaneous wind speed) for the apple price (the 5th row and 4th column in Fig. 6), all the factors showed the highest correlation values when there was a proper value of time delay d>0. In other words, almost no factors showed the highest correlation with the price when there is no time delay (i.e., d=0). Furthermore, with no time delay, several factors, such as *Sunsh* (i.e., the amount of sunshine) for tomato price and *Snow_∗* (i.e., the amount of snowfall) for apple price, showed nearly independent relations (i.e., close to zero correlation values) with the prices. This result indicates that it would be recommended to model the relationships between the weather and price patterns by taking the time delay into account. Even so, a careful interpretation of this result is needed. This result corroborates the necessity of time delay for modeling, as expected. However, the cross correlation (Equation (1)) is a linear function and thus can only say whether the sequences are linearly correlated or not. Therefore, it is difficult for the cross correlation to provide the optimal value of time delay for the relationships between the weathers and prices, because these relationships are generally expected to be complex nonlinear patterns. Regardless of the correlation coefficients, the time delay between the volume and price to one day was fixed (i.e., predicting the price at time t with the volume at time t−1). This setting is based on the following assumption that the volume on a certain day immediately affects the prices on several foreseeable future days, unlike the environmental conditions.

### 2.5 Model architecture

The LSTM [37] was adopted, a variant of traditional recurrent neural networks (RNNs) to capture long-term time-dependency by introducing a gating mechanism that controls the information flow to mitigate the long-term dependency problem [38,39]. An LSTM unit (called *memory*) consist of a memory cell, an input gate, an output gate, and a forget gate.

**Fig. 7:**
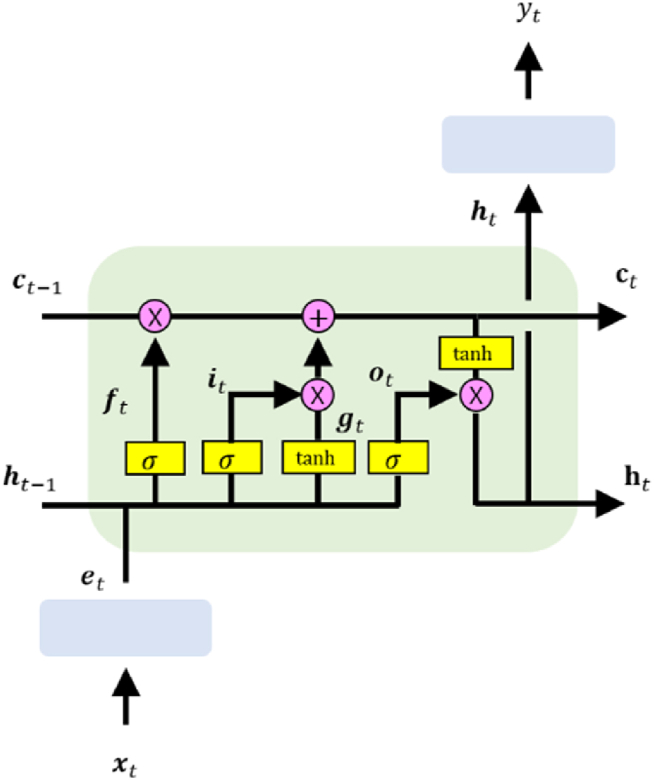
The overall architecture of our model.

Our model comprises a stack of an input embedding layer (for stable dropout at input-level; Equation (1)), a 1-layer LSTM (Equations (2), (3), (4), (5), (6), (7), (8))), and a regression layer (Equation (9)). Let $X=\left(x_{1},\ldots ,x_{T}\right)\in R^{T\times D}$ be a sequence of T feature vectors (e.g., a sequence of environmental conditions). For each time step t, the regression output $y_{t}\in R$ (e.g., price) from the $x_{t}\in R^{D}$ is computed as follows (note: the time delay d is omitted in Equations (2), (3), (4), (5), (6), (7), (8), (9)) for brief explanation):

(2) $$e_{t}=D\left(\text{GELU}\left(W_{e}x_{t}+b_{e}\right)\right)$$

(3) $$i_{t}=\sigma \left(W_{ie}e_{t}+U_{ih}h_{t-1}+b_{ih}\right)$$

(4) $$f_{t}=\sigma \left(W_{fe}e_{t}+U_{fh}h_{t-1}+b_{fh}\right)$$

(5) $$g_{t}=\tanh\left(W_{ge}e_{t}+U_{gh}h_{t-1}+b_{gh}\right)$$

(6) $$o_{t}=\sigma \left(W_{oe}e_{t}+U_{oh}h_{t-1}+b_{oh}\right)$$

(7) $$c_{t}=f_{t}⨀c_{t-1}+i_{t-1}⨀g_{t}$$

(8) $$h_{t}=o_{t}⨀\tanh\left(c_{t}\right)$$

(9) $$y_{t}=w_{y}^{T}D\left(h_{t}\right)+b_{y}$$

where $i_{t}$, $f_{t}$, $g_{t}$, $o_{t}$ are the input, forget, cell, and output gates at time t, $c_{t}$, $h_{t}$ are the cell and hidden states at time t, D(⋅) is the dropout operator, GELU(⋅) is the function of Gaussian error linear units [40], $W_{e}\in R^{E\times D}$ and $b_{e}\in R^{E}$ are the parameters for the input embedding layer, $\left\{W_{*e}\in R^{H\times E},U_{*h}\in R^{H\times E},b_{*h}\in R^{H}\right\}$ are the LSTM parameters for the corresponding gate ∗, σ(⋅) is the sigmoid function, tanh(⋅) is the hyperbolic tangent function, ⨀ is the element-wise multiplication, and $\{w_{y}\in R^{H},b_{y}\in R\}$ are the parameters for computing $y_{t}$. The input gate $i_{t}$ controls the information flow from the current input $e_{t}$ to the current cell state $c_{t}$. The forget gate $f_{t}$ adaptively forgets or remember the previous information from the previous state $h_{t-1}$. The cell gate $g_{t}$ corresponds to the candidate cell state at the current time step. The output gate $o_{t}$ controls the information flow from $c_{t}$ to $h_{t}$. The cell state $c_{t}$ stores the long-term temporal information over arbitrary time intervals. The hidden state $h_{t}$ corresponds to the output of LSTM cell at t. Fig. 7 shows the overall architecture of our model.

T=30 (e.g., predicting the price with a sequence of 30-day span weathers) and E=H=64. The dropout probability was set to 0.2 for both the input embedding and output layers. In this work, the model output for each input sequence X of length T corresponds to the regression output $y_{T}$ at the last time step T.

In practice, varying time delays d∈[0,180] for modeling was considered, as mentioned in the section of Statistical analysis for time delay. Let $y=f_{model}\left(X\right)$ be the operation of our model, corresponding to the equations from (2), (3), (4), (5), (6), (7), (8), (9). With an arbitrary time delay of d, the model takes the sequence of environmental conditions $X=\left(x_{s+1},\ldots ,x_{s+T}\right)\in R^{T\times D}$ of length T starting from an arbitrary time step s+1, and predicts the price $y_{s+T+d}$ at the time step of s+T+d. Meanwhile, when the trading volume is additionally given as input, the time delay $d_{v}$ between the volume and price to $d_{v}=1$ was fixed, regardless of the value of time delay d between the environmental conditions and price, as explained above. Notice that $d_{v}=0$ means the prediction of the price at time t with the volume corresponding to the same time t. This is an unrealistic setting for prediction, because both the volume and price on the same day t are determined after the trading on day t is finished.

For the model optimization, mean absolute error (MAE) was used as the loss function, as in Equation (10):

(10) $$l_{i}=\left|y_{i}-{\hat{y}}_{i}\right|$$

where $l_{i}$ is the loss for the i-th training sample sequence and $y_{i}$, ${\hat{y}}_{i}$ are the prediction (i.e., model output) and observation (i.e., ground-truth label) for the corresponding i-th input sequence, respectively. Rectified Adam [41] was used, with the learning rate of $10^{-3}$ and weight decay of $10^{-5}$, to optimize the above loss. All models were trained for 100 epochs with the batch size of 64. Five independent replicas of each experimental condition with different random seeds for reporting the average predictions were run.

For the evaluation metric, Nash–Sutcliffe model efficiency coefficient (NSE) was used [39,42], widely used in Hydrology. It is equivalent to the coefficient of determination ($R^{2}$), defined in Equation (11):

(11) $$NSE=1-\frac{{\left({{\hat{y}}_{t}-y}_{t}\right)}^{2}}{{\left({\hat{y}}_{t}-\bar{y}\right)}^{2}}$$

where $\bar{y}$ is the mean of observations. The NSE value is in the range (−∞,1]. The higher the NSE, the better the prediction accuracy.

The LSTM model was implemented using PyTorch [43].

## 3 Results

### 3.1 Performance for price prediction

**Fig. 8:**
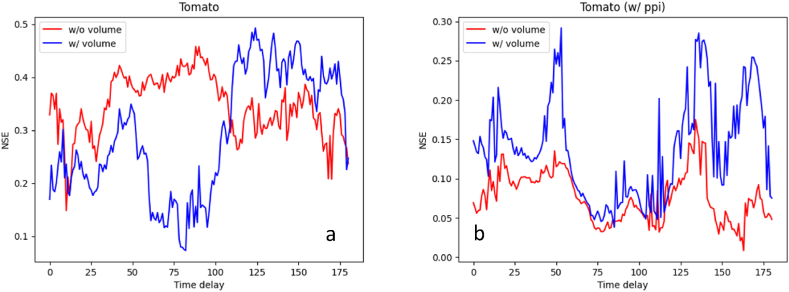
The results of tomato price prediction with all the environmental factors, excluding (a) and including (b) producer price index into the prices, respectively. Time delay: time delay between independent variable (environmental factors) and dependent variable (tomato price), day; NSE: Performance evaluation metric.

Fig. 8 shows the NSE of tomato price predictions according to the time delay, (a) nominal and (b) real-term prices, respectively. For the nominal price (Fig. 8a), the opposite patterns were observed depending on the existence of volume. Without the volume (red line), the best NSE of 0.458 was observed at d=88. The higher NSEs were observed in the range d∈[60,100] compared to other ranges. On the contrary, with the volume (blue line), a significant decline in NSE was observed in that range d∈[60,100]. The best NSE of 0.495 was observed at d=124. Besides, there is no significant difference in the best NSE (i.e., 0.458 vs 0.495; 0.2 %) depending on the volume.

For the real-term price (Fig. 8b), the overall NSEs were significantly lower than those for the nominal price. Nonetheless, the NSE trends according to d were similar regardless of the usage of the volume. They showed higher NSEs when d was around 50 or 130 than any other d values. Without the volume (red line), the best NSE of 0.175 was observed at d=134, where the NSE reduction compared to the nominal price was 0.283. With the volume (blue line), the top-2 NSEs were 0.292 at d=53 and 0.285 at d=136, where the NSE reduction compared to the nominal price was 0.203. In terms of the volume, the best NSE relatively increased 66.9 % when deploying the volume into the modeling of the real-term price (i.e., from 0.175 to 0.292). It means that the volume is a significant factor to predict the real-term prices of tomato, which is accordance with the fact that tomatoes have a relatively short storage period after harvest so are shipped to the market almost immediately. Under this condition, the volume could be a dominant factor in the price. Given that 1) the consistent trends were observed with respect to d regardless of the volume and 2) the impact of volume in modeling is in line with well-known fact, modeling the real-term price could bring more robust results than that of the nominal price, the overall NSEs for the real-term price were significantly lower than those for the nominal price though.

**Fig. 9:**
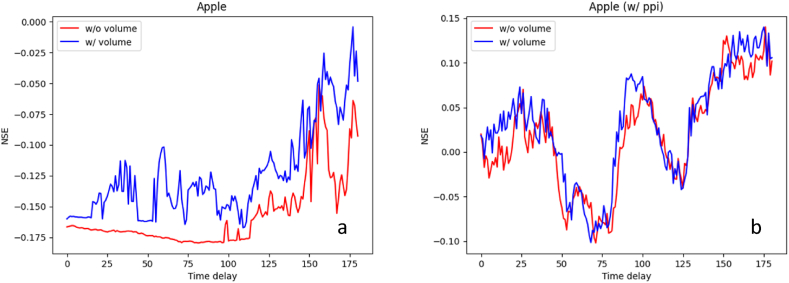
The results of apple price prediction with all the environmental factors. a: excluding producer price index; b: including producer price index. Time delay: time delay between independent variable (environmental factors) and dependent variable (apple price), day; NSE: Performance evaluation metric.

Fig. 9 shows the NSE of apple price predictions according to the time delay, nominal (a) and real-term prices (b), respectively. Regardless of compensating for the price with the PPI, the NSEs showed similar patterns both with and without the volume. However, all the prediction performances for apple were significantly poor compared to those for tomato. For the nominal price (Fig. 9a), the best NSEs were −0.051 (at d=156; red line) and −0.004 (at d=177; blue line) with and without the volume, respectively. For the real-term price (Fig. 9b), the same best NSE of 0.140 was observed at d=175 and d=176 with and without the volume, respectively.

The main differences of apple prices from tomato prices are two things. First, the apple price is scarcely affected by the volume. A possible explanation for this result is that apples have a much longer storage period than tomatoes. It means that the apple prices could be less sensitive to the volume over the past few days, as apples are generally more well-stocked than tomato. Moreover, as explained in the section of Price data, there are more missing values in the apple volume data than tomato volume data. In this case where many missing values are replaced by the same meaningless value (e.g., zero), model could prone to be trained toward the reduction of utilizing these trivial values. Second, the environmental conditions seem to have a low impact on apple price predictions. We will discuss this issue in the section of Discussion.

### 3.2 Explanatory power of price

**Fig. 10:**
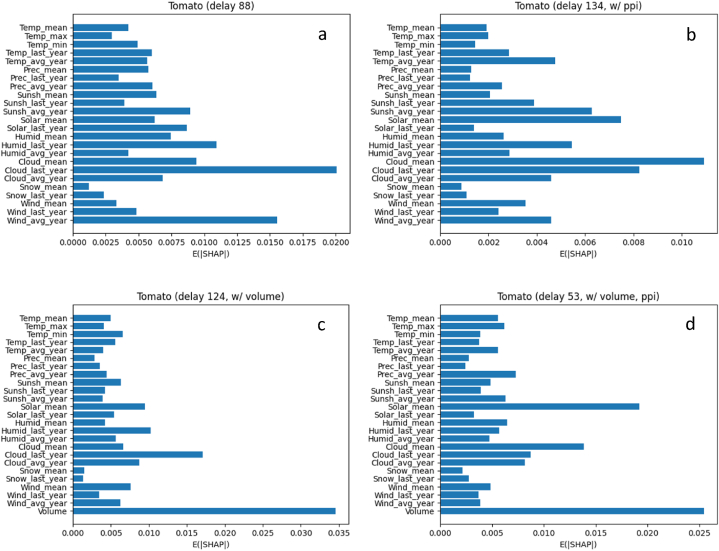
The influence of each independent variable, without (a & b) and with (c & d) the volume, on the tomato price at the delay showing the best NSE: nominal (a & c) and real-term prices (b & d), respectively.

**Fig. 11:**
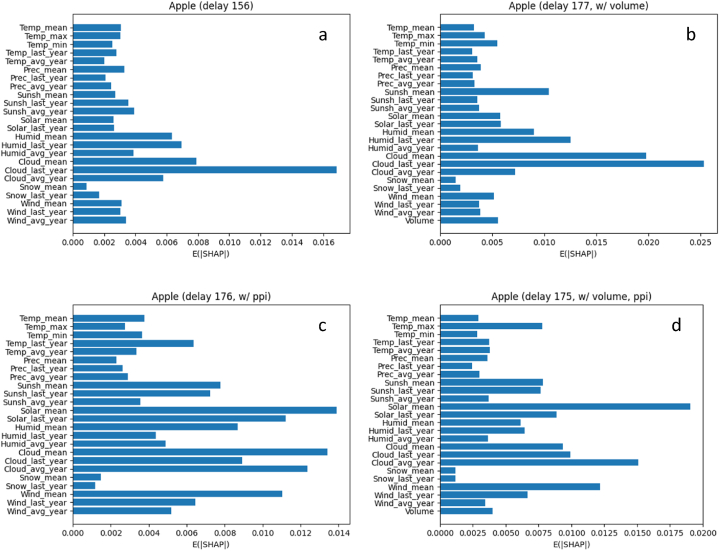
The influence of each independent variable, without (a & b) and with (c & d) the volume, on the apple price at the delay showing the best NSE: nominal (a & c) and real-term prices (b & d), respectively.

Fig. 10a–d and 11a-d explain the influence of each input variables (i.e., environmental factors and/or volume) on the output variable (i.e., price), for tomato and apple, respectively. The impact is represented by the average of absolute SHAP values, calculated using the SHAP methodology [44]. Note that the result in Fig. 10a–d and 11a-d does not tell us the direction of each independent variable with respect to the dependent variable: whether positive or negative relations. The analysis in this section is based on the mean of each environmental condition.

Among environmental conditions, the cloud amount consistently showed significant impacts, regardless of the fruit and price types. On the contrary, the amount of snowfall has little effect on the price predictions. For tomatoes (based on the real-term prices that showed robust trends), the quantity of solar radiation also has considerable importance to the prediction. The humidity and temperature showed lower but cannot be ignored importance. Like the amount of snowfall, the precipitation showed little impact. For apples, the quantity of solar radiation showed a large difference depending on the type of price (i.e., nominal vs real-term prices), where it showed a significant impact on the real-term price of apple. The other environmental factors showed low levels of the importance, which is accordance with the result presented in the section of Performance of price prediction (Fig. 9).

In terms of the volume, it has the best impact on the price predictions for tomatoes. No environmental conditions showed higher importance than the volume. The reason for this result is believed to be that environmental conditions are indirect factors for the prices, but the volume is more directly related to the prices, at least for tomatoes. For apples, on the contrary, the volume did not show a dominant impact on those for apples. These results of volume for both tomatoes and apples are in line with those presented in the section of Performance of price prediction (Fig. 7, Fig. 8), by supporting our explanations about the relationships among the volume, storage, and price, suggested in that section.

## 4 Discussion

Often, when climate change is discussed, rising air temperatures are cited as an example. And the rising air temperature is more dramatic in greenhouses. The maximum air temperature in South Korea is about 35 °C, but the air temperature in a greenhouse on July may reach to 40 °C, and it affects the crop growth and productivity [45,46]. Therefore, the air temperature may be a major factor in determining the price. This seemed to be proven in Fig. 4, but Fig. 4, Fig. 6 showed that the solar radiation had a greater effect on tomato price. Greenhouse heating caused by solar radiation results in rising air temperatures in greenhouses and reduces crop production [47]. Thus, farmers shade their crops under strong solar radiation and high air temperature conditions in summer, but the shading can reduce the photosynthetic efficiency due to light reduction. It is the same logic that cloud amount has a great influence on the prices (Fig. 4, Fig. 6). As photosynthetic efficiency decreases, fruit production also decreases, which ultimately may affect the prices. However, apple prices have less environmental impact than tomato prices, because apples take longer time to produce fruit than tomatoes.

Reduced production due to inappropriate climate causes problems from the flower development stage [48]. reported that as the air temperature rises by 4^o^C from 14^o^C to 26^o^C, flower development time of tomatoes becomes shorter, but the number of set fruits decreases. If the time is shortened, pollination and fertilization become unfavorable, resulting in fewer fruit number and smaller fruit size. On the other hands, apples need a certain level of low air temperature for flowering, but high air temperature reduces the number of bloomed flowers, affecting the fruit yield [49,50]. Moreover [51], reported that the fresh weight of ‘Fuji’ apple fruit in South Korea tended to decrease when air temperature changed from −15.0–35.9^o^C to −13.0–40.9^o^C during the growing season. A series of these results indicate that increased solar radiation due to climate change increases the air temperature in greenhouses, causing a decrease in the productivity of tomato and apple fruits, ultimately the fruit prices increase due to a decrease in quantity can be expected.

The results of tomato (Fig. 5) and apple (Fig. 6) price prediction with all the environmental factors indicated that currently established fruit prices are influenced by the environment from two to six months ago. In addition, the period takes longer for apples than for tomatoes. The reason for the situation can be found in the difference in the fruiting period of the two fruits. Generally, tomatoes are harvested about three months after sowing and apples are harvested in the autumn. Thus, it was confirmed that the environmental factors that affected the crop growth during the period sequentially affected the flowering and fruiting that occurred later. Although there are few similar studies on these results, in the case of leafy vegetables [52], reported that Kimchi cabbage and radish prices are directly affected by drought, abnormally high air temperature, abnormally low air temperature, and heavy rain. Since most leafy vegetables are harvested from the entire plant, they are inevitably more directly affected by the environmental factors than fruits. In addition, the harvested part of leafy vegetables is the source, and the harvested part of fruit vegetables is the sink. Since the sink is greatly affected by nutrients coming from the source, the sink may be affected if the environmental factors affect the source [53,54]. This is the reason it is hard to conduct these studies on fruit vegetables.

This study has shed light on the impact of climate conditions on the prices of tomato apple and have provided the insights using data-drive manners, such as deep learning. Nonetheless, there are several limitations to be addressed in future, all of which boil down to the data scarcity. Both the environmental and price data used in our experiments were collected only from South Korea. This means that the results derived here is limited to the local area, not a global one. In terms of data attribute, there is no factors in the data used that directly impact on the price. The climate conditions are indirect factors, so there is a limitation on the prediction accuracy when modeling prices with those indirect factors. We suppose that there are several hidden factors not available on data yet. Obviously, climate conditions affect the quality of fruits and accompanying prices. But in detail, it is more reasonable to consider that climate conditions affect several hidden factors that have more direct relationships to the quality, such as soil conditions, and then these hidden factors affect the quality. Of course, there could be several sub-relationships between hidden factors from the climate condition to the quality. Thus, it is important for further clarifying the weather-price mechanisms to discover what the dominant hidden factors are and how they interact. Therefore, beyond the environmental conditions, more various kinds of data related to cultivations should be collected to fully utilize recent data-driven technologies.

In terms of model architecture, the LSTM was used, a variant of RNN, as our prediction model. This issue is related to inductive bias. Note that inductive bias is a set of assumptions that an algorithm has for better explanation of data. A strong inductive bias could bring stable performances even with a relatively small amount of training data, only if the biases and data properties are well matched. In other words, it generally yields underfitted results (i.e., poor performances on both seen training and unseen test samples) when modeling data whose properties are far from the model assumptions [55]. RNNs are designed with a strong inductive bias of sequentiality, where the hidden state at each time step is computed from the current input and the previous hidden state [56]. It means that RNNs consider the later frames (in a given input sequence of frames) are considered more important than the earlier ones. However, this inductive bias is not always optimal for all kinds of time-series data. In view of our task, where predicting prices using climate conditions with fixed delay d, the price at time t+d is not always most significantly influenced by the environmental conditions at time t. Transformer [57] and its variants, having weaker inductive biases and thus providing more flexible time-series modeling than RNNs, could be an alternative to RNNs. But those generally requires much more training data due to its weak inductive bias, which is difficult to be satisfied under at least our experimental conditions.

Analyzing the relationship between environmental factors and agricultural commodity prices is a crucial research task. Based on this study, this study helps farmers and consumers comprehend how environmental factors influence levels in agricultural commodity prices. Predicting agricultural commodity prices offers vital information to both agricultural producers and consumers, contributing to the establishment of stable market conditions [1]. Furthermore, understanding environmental factors can reduce uncertainties and risks for agricultural production [58,59]. Evaluating these uncertainties and risks, and devising strategies for how agricultural and economic stakeholders can respond, is of utmost importance. The uncertainties arising from climate change can make predicting crop production challenging, potentially leading to increased volatility in agricultural commodity prices [60]. Therefore, the insights gained from this study can contribute to the formulation of agricultural and economic policies. By implementing appropriate responses and measures, Price or policy makers can enhance the predictability of agricultural commodity prices and explore ways to mitigate the risks associated with climate change. This, in turn, contributes to maintaining a sustainable agriculture sector and a stable market for agricultural commodities.

## 5 Conclusion

Agricultural product prices are affected by a wide range of variables, including cultivation environments, crop yield, consumer preferences, and macroeconomic changes such as inflation and international trade. These prices exhibit competitive, seasonal, and volatile characteristics due to the perishability of agricultural products, high logistics costs, and the inelastic nature of demand. However, as mentioned in the introduction, there are some examples of using machine learning simply to predict prices of agricultural products or to detect crop growth and diseases, but the use of machine learning in predicting prices of agricultural products based on various factors has not been common. The study quantitatively examined the relationship between environmental factors and agricultural product prices, identifying time lags between these variables. Through machine learning techniques, the study demonstrated the feasibility of predicting prices based on environmental impacts, offering valuable insights for reducing economic uncertainties. These findings can assist agricultural producers and policymakers in making informed decisions, stabilizing markets, and enhancing food security. Future research should expand the dataset to include diverse regions and crops while applying more sophisticated machine learning models to improve prediction accuracy. Integrating real-time data streams and exploring hybrid models that combine machine learning techniques with traditional econometric models are also essential future research directions to enhance prediction accuracy. Moreover, in-depth analysis of the relationship between climate change and agricultural prices could inform strategic policy development to strengthen food security and market efficiency. This study contributes foundational knowledge to support sustainable development in agricultural economics and food security through data-driven approaches.

## CRediT authorship contribution statement

**Sunghyun Yoon:** Writing – review & editing, Writing – original draft, Visualization, Validation, Supervision, Software, Methodology, Investigation, Formal analysis, Data curation. **Tae-Hwa Kim:** Writing – review & editing, Writing – original draft, Validation, Supervision, Resources, Methodology, Investigation, Formal analysis, Data curation, Conceptualization. **Dong Sub Kim:** Writing – review & editing, Writing – original draft, Visualization, Validation, Supervision, Resources, Project administration, Investigation, Funding acquisition, Conceptualization.

## Informed consent

Informed consent was obtained from all individual participants included in the study.

## Ethics declarations

Review and/or approval by an ethics committee was not needed for this study.

Informed consent was not required for this study.

## Data availability statement

Data associated with the study has not been deposited into a publicly available repository, the raw data supporting the findings of this study are available from the corresponding author at request.

## Funding

This work was supported by 10.13039/501100014189Korea Institute of Planning and Evaluation for Technology in Food, Agriculture and Forestry (10.13039/501100014189IPET) and Korea Smart Farm R&D Foundation (KosFarm) through Smart Farm Innovation Technology Development Program, funded by 10.13039/501100003624Ministry of Agriculture, Food and Rural Affairs (10.13039/501100003624MAFRA) and 10.13039/501100014188Ministry of Science and ICT (10.13039/501100014188MSIT), 10.13039/501100003627Rural Development Administration (10.13039/501100003627RDA) (RS-2024-00400011) and this work was supported by the research grant of 10.13039/501100002510Kongju National University Industry-University cooperation foundation in 2024.

## Declaration of competing interest

The authors declare that they have no known competing financial interests or personal relationships that could have appeared to influence the work reported in this paper.
